# Top 100 #PCOS influencers: Understanding who, why and how online content for PCOS is influenced

**DOI:** 10.3389/fendo.2022.1084047

**Published:** 2022-12-07

**Authors:** Maiar Elhariry, Kashish Malhotra, Michelle Solomon, Kashish Goyal, Punith Kempegowda

**Affiliations:** ^1^ College of Medical and Dental Sciences, University of Birmingham, Birmingham, United Kingdom; ^2^ Department of Medicine, Dayanand Medical College and Hospital, Ludhiana, Punjab, India; ^3^ UCL Division of Medicine, University College London, London, United Kingdom; ^4^ Department of Medicine, Delhi Heart Institute and Multispeciality Hospital, Bathinda, Punjab, India; ^5^ Institute of Metabolism and Systems Research, University of Birmingham, Birmingham, United Kingdom; ^6^ Queen Elizabeth Hospital, University Hospitals Birmingham National Health Service (NHS) Foundation Trust, Birmingham, United Kingdom

**Keywords:** polycystic ovary syndrome, PCOS, social media, influencers, high-income countries, low- and middle-income countries

## Abstract

**Background:**

With the exponential increase in digital space of social media platforms, a new group called social media influencers are driving online content of polycystic ovary syndrome (PCOS) which eventually influences behaviour and decision-making process. The objective of this study was to identify the top 100 social media (Twitter) influencers and organizations from across the globe who are advocating for PCOS. We further explored the origin and journey of these social media influencers.

**Methods:**

We identified the top 100 PCOS influencers and organizations between July and August 2022 using three social network analysis tools- Cronycle, Symplur and SocioViz. These influencers were invited to a semi-structured interview to explore why they chose to become an influencer and the support they have to deliver their online content. Two independent authors coded the anonymised transcripts from these interviews and broad themes were identified by thematic inductive analysis.

**Results:**

95.0% of individual influencers and 80% of organisations are from high-income countries. Most influencers in our study agree that social media is an essential tool in the present day to raise awareness of PCOS. However, they reiterated social media also has significant disadvantages that require consideration and caution. Most influencers were driven by poor personal experience and worked voluntarily to reduce misinformation and improve the experiences of women diagnosed with PCOS in the future. Although there is an interest in working together, there is currently minimal collaborative work between influencers.

**Conclusion:**

There is a global inequity of #PCOS influencers online. Establishing standards and support based on evidence may help develop more influencers, especially in low- and middle-income countries, so we can counter misinformation and provide locally acceptable guidance.

## Introduction

Polycystic Ovary Syndrome (PCOS) is one of the most common hormonal disorders affecting women accounting for 0.43 million disability-adjusted life-years from 1.55 million incident PCOS cases (1, 2). Latest studies have shown PCOS is no longer a condition affecting only the reproductive age women but a lifelong condition with increased risk for diabetes liver disease, endometrial cancer, obstructive sleep apnoea and impact on emotional wellbeing (3).

Social media has emerged as one of the largest medium through which people share and receive information.(Tao, Yang, and Feng 2020) Its greatest impact was seen through the COVID-19 pandemic when the public opinion was swayed based on the information shared online.(Qorib et al., 2023) Therefore, it is important that credible information is shared, not only with the people of PCOS, but also with the general public to create a positive and caring global community that understands the social aspects of PCOS and does not stigmatize women suffering from this condition. With the exponential increase in digital space of social media platforms, a new group called social media influencers (SMIs) have emerged (4). With growing literature on how the influencers impact behaviour and decision-making process (5–7), it is crucial to identify PCOS influencers. While there is literature about trends in social media influencers in surgical specialities (8–10), similar studies in PCOS are not available. Therefore, we conducted this study to establish the demographics and experiences of the top PCOS influencers.

## Materials and methods

We conducted this study from June to August 2022. The list of top 100 PCOS influencers and organizations was extracted from Cronycle (Right relevance API).(“Market Intelligence & Competitor Monitoring Software | Cronycle” n.d.) Cronycle uses a proprietary algorithm to generate a Twitter topic score for both people and organizations based on their engagement to determine the overall “influence” of a Twitter account within a topic of discussion. By leveraging machine learning, semantic analysis and natural language processing, Cronycle utilises graph partitioning techniques to determine a numerical score of “influence” based on connections (follower/following) to other influencers on a particular topic and secondarily by engagement (views, likes, retweets) which represents the authority of an influencer within the topical community (11). This also has been used in similar studies in other specialties like cardiology (12) and critical care medicine (13). Recently, some of the authors of this article have applied this methodology to study the global impact of stroke awareness month (14), deep vein thrombosis awareness month (15), hernia awareness Month (16) and world hypertension day (17). The alternative forms of the term “Polycystic Ovary syndrome” that were included in the search query on Cronycle were- polycystic ovary, sindrome de ovario poliquistico, pco-syndrom, hyperandrogenic anovulation, syndrome des ovaires polykystiques, polycystic ovarian syndrome, pos, stein-leventhal syndrome, síndrome dos ovários policísticos, pcos, polycystic ovary syndrome (japanese), syndrome delle ovaie policistiche, polycystic ovary disorder, Polycystic Ovary Syndrome (chinese).

We contacted the top 100 Twitter PCOS influencers to take part in our structured interview sharing their experiences regarding PCOS. Their contact details were obtained from publicly accessible professional profiles and one follow-up email was sent to the non-respondents. To limit bias associated with a single tool, we also invited top influencers identified through Symplur (18)and Socioviz (19). These software have different approaches to identify the top influencers. SocioViz calculates the top Influencers based on the number of retweets and mentions received in the set timeframe. Based on the quality of the number of mentions received, Symplur uses machine learning to identify the top influencers. A mention’s quality is determined by its influence, its healthcare stakeholder status, and its overall influence in healthcare social media. It is done to minimize the manipulation of simplistic metrics, such as mentions, tweets, followers, etc.

Upon accepting our invitation, we invited the influencers for a 15-minute semi-structured interview at the time of their convenience. With their consent, the meeting was recorded with auto-transcript feature to have a written transcript of the conversation that was later used for thematic analysis. The interview questions are listed in **Supplementary 1**.

Influencers were requested to not turn on their camera, share any patient details, or any other information they were not comfortable sharing. The context of the questions was broadly shared with the interviewee beforehand in the invite email and the interviewee reserved the right to not answer any specific question throughout the interview. At the beginning of the interview, the interviewee was requested to consent to the usage of the interview data for our study. The transcripts of all conversations were saved anonymously without the name of the interviewee in a password-protected encrypted folder. The mode of personal interview was chosen over an online survey to have more individualized and specific answers as most of the questions were open-ended and thematic in nature. The study was approved by the ethics committee of Delhi Heart Institute and Multispeciality Hospital, Punjab, India.

The anonymised transcripts were coded by 2 independent authors by thematic inductive analysis using NVivo and broad themes were identified depending on the interview data. Codes generated were merged and grouped into subcategories. Furthermore, the demographics data of top influencers were studied based on their profession and country of residence.

## Results

**Figure 1:**
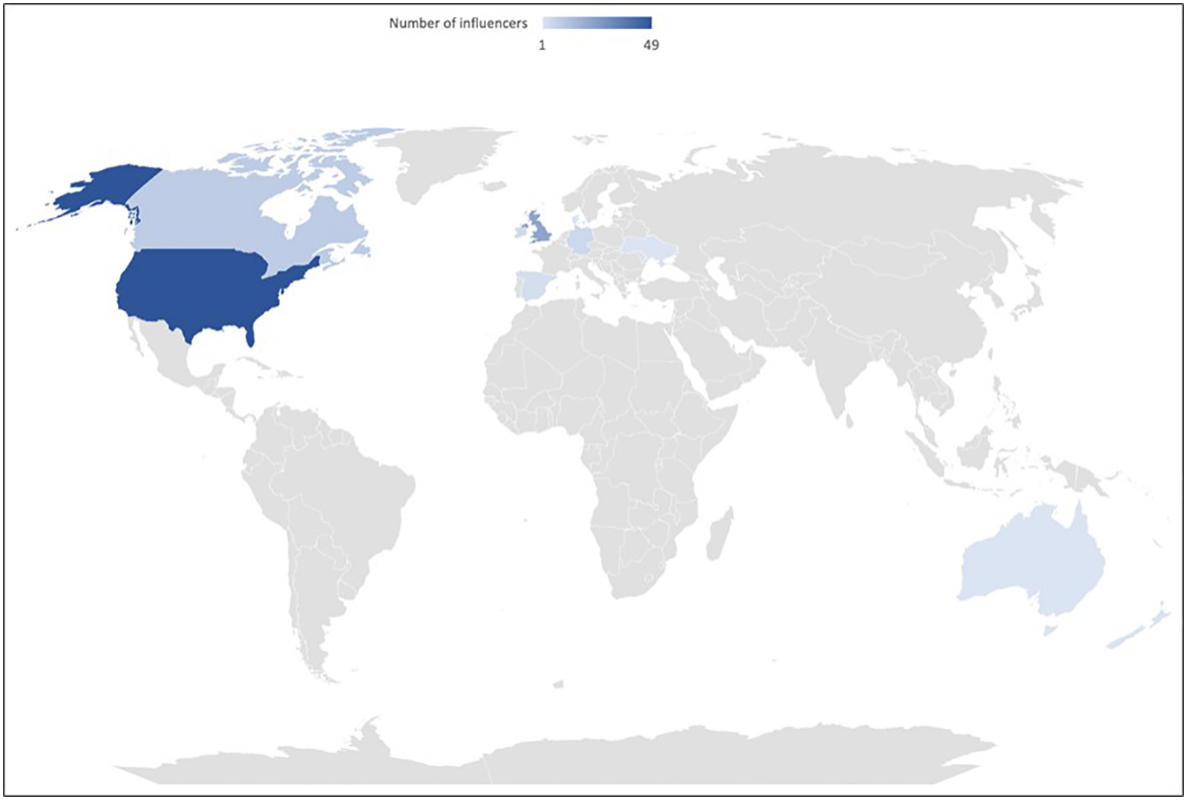
Geographic spread of top 100 influencers for PCOS. The world map is for diagrammatic representation only and doesn't purport to be the political map of any country.

The top 100 individual and organisation influencers for #PCOS is listed in **Tables 1**, **2** respectively. Of the top 100 individual influencers, 73.2% (71) were female and 26.8% (20) male; 3 individuals’ gender was unknown. 95% of influencers were identified to be from high income countries. One influencer was from low- and middle-income country (LMIC). We could not identify the country of residence for four influencers (**Figure 1**) (21). Furthermore, the top 3 countries of residence were the USA (n=49), UK (n=22) and Canada (n=9).

**Table 1:** Top 100 influencers for PCOS on social media (twitter).

Name	Username	Followers	Occupation	Country
James DiNicolantonio	drjamesdinic	105884	Medical doctor & author	United States
Dr. Jennifer Ashton	DrJAshton	93980	Obstetrician & gynaecologist	United States
Ted, ö°Ô∏è Naiman	tednaiman	85040	Diet and exercise doctor	United Kingdom
Samantha Kelly	Tweetinggoddess	67388	Business consultant	Ireland
Dr. Martha Gulati, ÄúGet Vaccinated Please, Äù	DrMarthaGulati	46000	Medical doctor: women’s health & heart disease	United States
Maria Emmerich	MariaEmmerich	22643	Nutritionist & writer	United States
Raewyn Teirney	DrRaewynTeirney	19385	Female fertility specialist	Australia
Rosey	PNDandMe	18088	Founder - PND Hour	United Kingdom
PCOSGurl (Ashley)	PCOSGurl	17865	Educator & patient advocate	United States
Dr. Doni Wilson	glutenfreedoc	16252	Naturopath & author	United States
PCOS Diva	pcosdiva	14223	Mentor	United States
Danielle Omar MS RDN	2eatwellRD	13563	Writer	United States
Carrie Diulus, M.D., FAAOS	cadiulus	12281	Orthopaedic spine surgeon	United States
Thom Singer, CSP üåü	thomsinger	12170	CEO - Austin Technology Council	United States
Dr. Poppy Daniels	drpoppyBHRT	10194	Obstetrician & gynaecologist	United States
Dr Annabel	SoSowemimo	9712	Sexual & reproductive health doctor	United Kingdom
Dr. Cheryl Arutt	drcherylarutt	9621	Clinical and forensic psychologist	United States
PCOS Awareness	AwarenessPCOS	9545	Activist	N/A
Adrian Segar	ASegar	8651	Meetings designer and facilitator	United States
P√°draic Gilligan	Padraicino	7786	Manager in travel consultancy	Ireland
Alan Stevens	mediacoach	7751	Speaking specialist	United Kingdom
Dr. Jolene Brighten	drbrighten	7739	Naturopath - endocrinology	United States
Linda Scruggs BSN, RN	UnboxedMom	7484	Reproductive endocrinology and infertility nurse	United States
Mark Gordon	MarkGordonMFE	7444	Event Director - consulting	United States
Felice Gersh MD	DrFeliceGersh	7082	Gynaecologist	United States
Jen Faulkner	jfaulknerwriter	7066	Writer	United Kingdom
Eliana Casta√ ± eda	elianasc_21	7045	Gynaecologist	Spain
Spin Doctor	SpinDr	7009	Reproductive law attorney	United States
Leah Campbell (she/her)	LeahWritesStuff	6836	Writer & editor	United States
Lara Briden	LaraBriden	6685	ND clinican - women’s health	New Zealand
, ú^®^ Elly , ú^®^	EarnestlyElly	6120	N/A	N/A
Jody Day (Gateway Women)	gatewaywomen	6074	Psychotherapt, writer & speaker	Ireland
Sara R. Cohen	fertilitylaw	6072	Fertility lawyer	Canada
Dr. Drai	DrDraiOBGYN	5966	Founder - magnify momentum	United States
Melanie Elliott	Mom2TLE	5478	Consultant - M3 development	United States
Julie Duffy Dillon (she/her)	FoodVoiceRD	5426	Podcast host	United States
Scott Isaacs, MD, FACP, FACE (he/him)	scottisaacsmd	5402	Endocrinologist	United States
Susan Dopart, RDN,CDCES	susandopart	5184	Dietitian nutritionist	United States
Mara Clarke	maraclarke	5110	Abortion supporter	United Kingdom
Pietro Bortoletto, MD	BortolettoMD	4854	Reproductive medicine doctor	United States
William Thomson	williamevents	4708	Events consultant	Spain
Dr. Pamela Frank, ND	PamelaTorontoND	4703	Naturopath- infertility	Canada
kate brian	katebrian	4416	Women’s health writer	United Kingdom
Sarah Holland	FertileMindset	4372	Podcast host	United Kingdom
Kimberley Logan	Fertility411	4306	Infertility patient advocate; founder of IVF center solutions	United States
Dr Anita Mitra	GynaeGeek	3883	Gynaecologist	United Kingdom
Mathew Leonardi MD PhD üè≥Ô∏è, Äçüåà	MathewLeonardi	3807	Obstetrics & gynaecology surgeon	Canada
The Next Family	thenextfamily	3781	Influencer	United States
Louise Perkins King	louise_p_king	3706	Surgeon & reproductive bioethics specialist	United States
Donielle Baker	donielle	3200	Writer	United States
Laura Spoelstra üá≥üá±üá¨üáß	Laura_Spoelstra	3143	Business owners coach	United Kingdom
Adam Balen	BalenAdam	3079	Professor of reproductive medicine	United Kingdom
Jenny Medlen	ActualJenny	3027	Writer	United States
Judy Kucharuk	judylaine	2881	Writer & blogger	United States
Mark Perloe	IVF_MD	2814	(retired) Reproductive endocrinologist	United Kingdom
Emma Cannon Fertile	emma_cannon	2751	Writer	United Kingdom
Natalya Mykhalko	mykhalko	2674	Writer	Ukraine
Martha McKittrick RD	McKittrickRD	2664	Dietitian	United States
Aaron R.Chidakel, MD	ACinNYC2K19	2629	Endocrinologist	United States
PCOS SUPPORT GIRL	PCOSsupportgirl	2545	Patient advocate	United States
Kerrin MacPhie	MICEkerrin	2524	CEO - Meetings Industry Association	United Kingdom
Karen Hobbs BA (Hons)	karen_hobbs	2470	Writer & comedian	United Kingdom
Sunny Days	sunnydayto	2389	N/A	United Kingdom
Dermot Ryan	MeetDermotRyan	2336	Account direction - KIT group GmbH	Germany
Dr Marjorie Dixon	DrMarjorieDixon	2324	Infertility doctor & gynecological surgeon	Canada
Hillary Wright	PCOSDiet	2302	Writer	United States
Tracey Sainsbury	IVFcounsellor	2115	Fertility counsellor	United Kingdom
Peter Cramer	erlebnispete	1999	Owner - MICE marketing	Germany
Joaquin Llacer	DrLlacer	1838	Reproductive medicine doctor	Spain
Nyx Cole	Nyxks	1826	Blogger	Canada
Our Misconception	rmisconception	1805	Writer & Women’s health advocate	United states
Fiona McCulloch ND	FionaMcnd	1793	Writer	Canada
Michelle Dipp	dipp	1700	Founder - Biosprins Partners	United Kingdom
Gabriele Schulze	GSchulzeBerlin	1551	Speaker, trainer and consultant	Germany
Dr Kylie Baldwin	DrKylieBaldwin	1549	Medical sociologist	United Kingdom
Dr. Eric J. Forman	EricFormanMD	1508	Medical & lab director - Columbia university fertility centre	United States
svend lindenberg	svli	1465	Director - Copenhagen fertility center	Denmark
Fertility Law Canada	sherrylevitan	1464	Fertility lawyer	Canada
Ricardo Azziz	ricardoazziz	1313	Medical doctor & Science and Strategy officer	United States
Corey Whelan	coreygale	1307	Health and wellness writer	United States
Renetta DuBose	RenettaReports	1289	Weekend anchor	United States
Chris Marquette	ChrisMarquette	1261	Vegetarian sports nutritionist	United States
Kate Davies - Fertility Nurse Consultant	fertjourney	1227	Fertility nurse consultant	United Kingdom
Carolyn Alexander	DrCAlexanderFer	1225	Fertility doctor	United States
Lisa Rosenthal	RosenthaLisa	1058	Patient advocate- reproductive medicine	United States
·ó^©^·ó∞II ü¶ñ	amii0484	1048	Student nurse	United Kingdom
Small Town Girl	MommaCanuck	973	Blogger	Canada
Lily Lai, PhD	Acuandherbs	903	Acupuncturist	United Kingdom
Dr. Hernandez-Rey	hernandezreyivf	872	Infertility doctor	United States
Your mom	frickfrackfrock	775	N/A	N/A
Drew Nesbitt R.TCMP	drewnesbitt	688	Acupuncturist & nutritionist - fertility specialist	Canada
Robin Writes Too	LicitRecidivist	669	Writer	United States
Peter Blach	peterblach	585	N/A	Germany
, Ä¢ Suzy , Ä¢	AwesomelyIced	523	N/A	N/A
Diana	OurExpandingZoo	498	N/A	United States
Lauren (she/her)	onfecundthought	444	Writer	United States
Trends for Events	trendsforevents	410	Events consultant	Germany
Deli	adelifish	354	Artist	Ireland
Davina Rudnick Fankhauser	DavinaAdvocate	341	Educator and fertility advocate	United States
Judy Simon MS RD	JSimonRD	278	Dietitian	United States

**Table 2:** Top 100 organisation influencers for PCOS on social media (twitter).

Name	Username (*CS)	Followers	Organisation type	Country
Vagina Museum	vagina_museum	139078	Charity & network	United Kingdom
RoyalCollegeObsGyn	RCObsGyn	48454	Professional body	United Kingdom
London & Partners Business	businesslondon	28358	News	United Kingdom
Endocrine Society	TheEndoSociety	27200	Charity & network	United States
Endometriosis.org	Endometriosis	23390	News	N/A (global)
Endometriosis UK	EndometriosisUK	22841	Charity & network	United Kingdom
Northstar Meetings Group	NorthstarMeets	22324	Business	United States
Monash FODMAP	MonashFODMAP	19527	HCP	N/A (global)
Period!	PeriodMagazine	17961	News	Netherlands
PCOS Nutrition Center |Angela Grassi	PCOSnutrition	17363	HCP	United States
Events Industry Council	Events_Council	16107	Business	United States
Vicious Cycle: Making PMDD Visible	messefrankfurt	16033	Business	Germany
Miscarriage Association	MiscarriageA	15134	Charity & network	United Kingdom
Endometriosis Foundation of America (EndoFound)	Endofound	15107	Charity & network	United States
LifeBoss Health	lucoleman	13527	HCP	N/A
Fertility Network	FertilityNUK	13294	Charity & network	United Kingdom
Fertility Road	FertilityRoad	12766	News	United Kingdom
Campaign Experience Awards	CxExperience	11834	Business	United Kingdom
European Society of Endocrinology (ESE)	ESEndocrinology	11394	Professional body	N/A (international)
The Meetings Show	MeetingsShow	11038	Business	United Kingdom
SITE	SITEGlobal	10994	Business	N/A (global)
Medscape Endo	MedscapeEndo	10761	News	N/A (global)
Speakers Corner	Speakers_Corner	10650	Business	United Kingdom
Executive Speakers	ExecSpeakers	9464	Business	United States
Glow, Inc.	GlowHQ	9012	Charity & network	United States
PCOS Challenge	pcoschallenge	8943	Charity & network	United States
National Speakers	NSBSpeakers	8327	Business	United States
Meetings Network	MeetingNetwork	8262	Charity & network	Canada
Elsevier	ObGynAdvance	8057	News	N/A (global)
Keppler Speakers	KepplerSpeakers	7967	Business	United States
World of DMCs	World_of_DMCs	7916	Business	N/A (global)
The FSRH	FSRH_UK	7496	Charity & network	United Kingdom
Endocrine Society Journals	EndoSocJournals	7472	News	United States
Ashfield Event Experiences	AshfieldEventEx	6759	Business	N/A (global)
Fertility Centers of Illinois	fertcentersofil	6748	HCP	United States
MCI for Associations	MCIAssociations	6595	Business	N/A (global)
Creating a Family	CreatingaFamily	6574	Charity & network	United States
Verity PCOS Charity	veritypcos	5960	Charity & network	United Kingdom
Master The Event	MasterTheEvent	5959	Business	United States
MPI UK & Ireland	MPIUKI	5930	Business	United Kingdom
SH:24	sh24_nhs	5771	HCP	United Kingdom
beam	WearebeamUK	5468	Business	United Kingdom
The Fertility Show	fertilityshow	5391	Charity & network	United Kingdom
Endocrine Connections	EndoConnect	5307	News	N/A (global)
CREATE Fertility	CreateIVF	5200	HCP	United Kingdom
AIME	AIMEAsiaPacific	4851	Business	Australia
Boston IVF	BostonIVF	4751	HCP	United States
New Hope Fertility	NewHopeFC	4737	HCP	United States
Shady Grove Fertility	SGFertility	4598	HCP	United States
Fertility Solutions	FertilityDocsNE	4469	HCP	United States
AIM Group Int	AIMGroupInt	4411	Business	N/A (global)
Natural Cycles	NaturalCycles	4325	HCP	N/A (global)
Worldwide EndoMarch	WWEndoMarch	4161	Charity & network	United States
Leading Authorities	LAIspeakers	4119	Business	United States & United Kingdom
Resolve New England	ResolveNewEng	4108	Charity & network	United States
Dr. Drai	viciouscyclepmd	4108	Patient group	N/A (global)
Jean Hailes	JeanHailes	4096	Charity & network	Australia
Congrex Switzerland	Congrex	3970	Business	Switzerland
Circle+Bloom	CircleBloom	3746	HCP	United States
Fertility Network	FNScotlandUK	3700	Charity & network	United Kingdom
Capitol City Speaker	CapCitySpeakers	3651	Business	United states
Kenes Group	Kenes_Group	3612	Business	Switzerland
Washington Fertility	FertilityWFC	3579	HCP	United States
GPJ UK	GPJExpLondon	3575	Business	United Kingdom & Norway
Fertility Matters Canada	FertilityMattrs	3480	Charity & network	Canada
Conference Partners	ConferencePart	3469	Business	United Kingdom & Ireland
H&E Fertility Centre	FertilityUnit	3361	HCP	United Kingdom
Conceivable Dreams	IVF4ON	3184	Charity & network	Canada
ISE	isendo	3004	Charity & network	N/A (global)
Healing Infertility	thefertilemind	2993	HCP	Canada
Fertility Centers of New England	fcneivf	2919	HCP	United States
Glasgow Convention Bureau	meetglasgow	2881	Business	United Kingdom
MCI UK	MCI_UK	2737	Business	United Kingdom
Lister Fertility	ListerFertility	2718	HCP	United Kingdom
ART of Infertility	artofif	2253	Charity & network	United States
BabyQuest Foundation	BabyQuestGrants	2175	Charity & network	United States
NGA Law	NGambleAssoc	2108	Business	United Kingdom
PCO Association Inc	pcoasn	2046	Business	Australia & New Zealand
Misconceived Films	_Misconceived_	1921	Business	Canada
Acacio Fertility	AcacioFertility	1861	HCP	United States
Genesis Fertility	GENESIS_NYC	1812	HCP	United States
COGI Congress	cogicongress	1809	Charity & network	N/A (global)
Abbey Conference	abbeyconference	1690	Business	Ireland
How to Buy a Baby	howtobuyababy	1654	Patient group	United Kingdom
Men Having Babies	MenHavingBabies	1654	Charity & network	N/A (global)
Egg Donor America	EggDonorAmerica	1575	HCP	United States
INCON Group	INCONGroup	1508	Business	Ireland
SoCal Reproductive	SCRCivf	1449	HCP	United States
FCC Sperm Bank	SpermBank	1415	HCP	United States
Frankfurt Convention	MeetFrankfurt	1354	Business	Germany
VOK DAMS worldwide	VOKDAMS	1324	Business	N/A (global)
lialo.com · Orte und ihre Geschichte(n) entdecken	lialo_com	1216	Business	Germany
Pride Angel	prideangel	1187	Charity & network	N/A (global)
Fertility Resources of Houston	FertilityResLLC	1090	HCP	United States
Maze Men’s Health	mazemenshealth	1090	HCP	United States
RMA Network	thermanetwork	1086	HCP	United States
CHR	CHRNewYork	1069	HCP	United States
Bride of Boogedy	Pregnant:Pause	1043	Patient group	N/A
The Surrogacy Group	SurrogacyGroup	1036	HCP	United States
Laurel Fertility	laurelfertility	980	HCP	United States

CS, Case Sensitive.

Amongst top 100 organisations, 80 worked in HICs and 18 worked internationally. None of the top organisation influencers (excluding two influencers from unknown locations) were from LMIC. The most prominent country of residence was the USA (38 organisations) followed by the UK (27 organisations). The organisations were classified as the following: Charities & Networks (n=25), patient support groups (n=3), Professional Bodies (n=2), News (n=8), Business (n=34) and Healthcare practices/professionals (n=28).

Of the total 100 influencers invited for an interview, 18 responded- eight agreed to meet and were interviewed, five agreed to meet but were not interviewed (due to loss of contact after agreeing on a day and time), five declined the invite (three were involved with nonmedical topics that are also abbreviated as PCOS. two explained that while they are may be linked to PCOS, their expertise in the field is limited).

Out of the top 100 PCOS influencers contacted, a total of 8 influencers completed the interview. The 5 major themes that emerged from the analysis were: “Influencer Traits’’, “Relation to PCOS”, “Social Media Content”, “Thoughts on Social Media”, and “Bringing Change”. The sub-themes of each of these are summarised in **Table 3**.

**Table 3:** Thematic analysis of the interviews of PCOS influencers.

Main theme	Sub-themes
Influencer Traits	Occupation, Partner Organisations
Relation to PCOS	PCOS Diagnosis, Fears around PCOS, Influencer’s Journey
Social Media Content	Platforms used, Influencer content
Thoughts on Social Media	Reasons for Using Social Media, Advantages, Disadvantages, Advice and precautions
Bringing Change	Empowering Patients, PCOS Awareness Month Activities

### Influencer traits

Of the 8 participants, five were based in England and three participants were based in the USA; five were health care professionals. Healthcare professionals were either researchers (2/5) or doctors (1 internal medicine and 2 metabolic endocrinology). The 3 other non-healthcare professionals had backgrounds in the fields of: psychology, management, and chemistry. Five Participants also demonstrated activity in other fields of endocrinology that they mentioned can be linked to PCOS. Amongst these fields, the most common ones were: “Obesity” (n=4), “Wellbeing” (n=4), and “Infertility” (n=3).

### Relation to PCOS

Different motivational and influential factors contributed to the participants decision to get involved with PCOS awareness. “Spread of misinformation” (n=7), “lack of support and correct information” available for women diagnosed with PCOS (n=6), “Misconceptions of PCOS impacts on health” (n=5), and “misconceptions on the ability to make changes to better one’s lifestyle” (n=5) were the most common reasons participants shared why they decided to become influencers. Six participants explained the responsibility they felt to support women with a PCOS diagnosis and reduce the subsequent uncertainty they experienced.

### Content

All participants reiterated the need to target a large proportion of the relevant audience by resorting to more than one social media platform. Five participants use twitter, three have a blog page, three use Instagram, and two rely on Facebook (some participants use more than one). Four participants created their own webpage. Other platforms that were mentioned include: TikTok (n=1), WhatsApp group (n=1) and clubhouse (n=1). Content that the influencers included focused on wellbeing (n=6), Lifestyle advice (n=4), recommended diets and nutrition (n=3), and influencer’s experiences in different aspects in relation to PCOS (n=3). Five participants explained that their content was personal to what they thought was relevant in their personal journey. Moreover, most participants highlighted the importance of “ensuring the information they publish is correct and accurate” (n=6).

We further studied the topics our eight interviewees mentioned they post on social media. There were 25 references on this topic. The 3 most common themes representing topics posted by the influencers were: Support groups (7 references), research and signposting (5 references), wellbeing and advice (4 references) and influencer journey (4 references). Other content includes: dietary advice and tackling myths/misunderstandings.

### Thoughts on social media

Half of the participants expressed that social media allowed easy and fast dissemination of information (4/8). Six participants explained that the main reason they use social media is to ensure they publish evidence-based information after expressing their concern over the large prevalence of incorrect information and conception. Two participants shared that they use social media as they find it easy to contact and collaborate with other organisations and influencers. Four participants used social media to support and advise women with PCOS facing challenges with their diagnosis. They referred to it as an attempt to create a “support network” and a “common platform” that women with PCOS can relate to and resort to. While all participants shared the perspective that social media decreased feelings of alienation and increased support, they were equally concerned about the misinformation and the need to combat it. All participants also disclosed that criticism and hate is a concern they have around using social media. Six participants had personally experienced criticism on social media.

### Bringing change

The main goal for all our participants was their desire to bring a change and empower women diagnosed with PCOS. Several suggestions were discussed in the interviews: to group all PCOS resources in one space so that it is easier for the target audience to access it (n=5), the importance of encouraging women with PCOS to make their own choices and lifestyle changes (n=5). Participants shared their plans for PCOS awareness month which included frequent blogs, lighting up a significant building in the city they are based in teal colour to increase PCOS awareness. All participants were open to collaborate and open to sharing resources on their platforms.

## Discussion

Our study, first of its kind, shows that #PCOS influencers is limited by geographical and ethnical diversity. Although other researchers investigated the use of social media to relay medical information about PCOS, we are the first to study the influencer or content-generators’ experience of using social media. We also show for the first time there are a variety of medical and non-medical organisations who influence the PCOS content online. This is important to collaborate and ensure evidence-based content is shared to minimise misinformation.

Most influencers in our study agree that social media is an important tool in the present day to raise awareness of PCOS. However, they reiterated social media also has significant disadvantages that require consideration and caution. Influencers highlighted the lack of support in their personal journey which may translate to the limited support available to PCOS patients as well as, stigma and fears that may be linked to receiving such a diagnosis in different age groups and demographics.

Our data shows the current social media landscape is mostly influenced by HICs and this may drive the content viewed and accessed by globally. Several researchers have established the racial and ethnic variation in the prevalence and severity of PCOS phenotypes (22, 23). There is also increasing evidence on the impact of PCOS on emotional wellbeing. Therefore, it is not unreasonable to draw inference that needs of people with PCOS vary across regions and ethnicity. Hence there is a need to encourage and empower influencers across the world to meet local demands.

In the current day and age, it is almost inevitable that social media and information conveyed through it carries a large weight and can reach a large range of audience (24). This gives content-creators large power in terms of their ability to influence social media users and their respective audience. However, there are no standards or regulations to create educational or influencing content currently. There is no reliable data available regarding the influencers and social media content creators in LMICs. Some organisations provide general guidance How to find reliable health information online (25, 26). Some have attempted to standardise the medical and scientific information available online (20). However, the huge number of new websites launched each year and expenses involved in validation has limited both the standardisation organisation and the influencers to achieve such a status.

A study by Saroja and Chandrashekar identified 15 websites in 2010 which provided information on PCOS. However, none of them met all the standard criteria for quality set by the authors (27). A study by Sanchez et al. exploring how online teen and women’s magazines portray women with PCOS showed articles depicted PCOS symptoms as a hindrance to women’s social roles as wives and mothers and largely placed personal responsibility on women to improve their health (28). Interestingly, experiences of Latina and African American women and adolescents with PCOS were also absent from these women’s magazine articles. These findings highlight the urgent need for establishing guidance, support and regulations to positively influence PCOS and limit misinformation on social media.

The main strengths of this study are the use of three independent software to identify top social media influencers and the open-ended discussion with the influencers enabling a wider range of input from participants. However, a low response rate for invitations might decrease the generalisation of the results. Nevertheless, many of the identified concepts were reiterated by multiple participants suggesting the need to improve the existing support for women diagnosed with PCOS. While we identified the topics posted by our influencers as described by them in the interviews, future work focussing on the actual social media content can help identify common topics that are being posted and discussed by influencers.

## Conclusion

There is a global inequity of #PCOS influencers online. Most influencers were driven by poor personal experience and work voluntarily to reduce misinformation and improve the experiences of women diagnosed with PCOS in the future. Although there is an interest to work together, there is currently minimal collaborative work between influencers. Establishing standards and support based on evidence may help develop more influencers, especially in the LMICs, so we can counter the misinformation and provide locally acceptable guidance.

## Data availability statement

The original contributions presented in the study are included in the article/**Supplementary Material**. Further inquiries can be directed to the corresponding author.

## Ethics statement

The studies involving human participants were reviewed and approved by Delhi Heart Institute and Multispeciality Hospital, Punjab, India. Written informed consent was not provided because Consent was obtained during the interview and recorded electronically and on video.

## Author contributions

ME and KM conducted the searches and screened the data from three social network analysis tools. ME and KM have been involved in all stages of the study, contributed equally to this work, and share the first authorship. MS identified the occupation and country of residence for the top 100 influencers and organisation type and country of location for the top 100 organisations for #PCOS. KG was involved during conceptualisation, finetuning the research methods and obtaining ethics approval. KM and PK conceptualised and supervised all stages of the study. PK and ME critically analysed the codes from interviews to arrive at appropriate themes for results. All authors agree to be accountable for all aspects of the work in ensuring that questions related to the accuracy or integrity of any part of the work are appropriately investigated and resolved.
