# Supplementary material for: Top 100 #PCOS influencers: Understanding who, why and how online content for PCOS is influenced
Source: Front Endocrinol (Lausanne). 2022 Dec 7;13:1084047. doi: 10.3389/fendo.2022.1084047 (PMC9768020; doi:10.3389/fendo.2022.1084047)
Supplement: Supplementary file 1 [file DataSheet_1.docx]

Supplementary Material

**Supplementary 1: The interview questions used for the study**

Tell us more about yourself, what you do and what is your involvement with PCOS?

If applicable, have you been clinically diagnosed with PCOS? If yes, then ask the following questions-

At what age were you diagnosed with PCOS?

What are your experiences on your journey toward PCOS diagnosis? This would include the symptoms, consultation, explanation, understanding, treatment, and current management of the condition.

How did you start your social media journey advocating about PCOS?

What are your experiences with PCOS awareness through social media? Which platforms do you use for this?

What do you feel are the current challenges specifically related to social media in destigmatizing PCOS and increasing awareness about PCOS?

Have you faced any kind of criticism regarding any social media post?

What are your expectations and plans for PCOS awareness month?

If we plan a combined event based on all the inputs we receive, would you be interested to promote and participate in it?
